# Retraction: Analysis of the key influencing factors of China’s cross-border e-commerce ecosystem based on the DEMATEL-ISM method

**DOI:** 10.1371/journal.pone.0362523

**Published:** 2026-11-11

**Authors:** The PLOS One Editors

The *PLOS One* Editors retract this article [1] due to concerns about:

- Citation of unretrievable references, specifically References 6, 16, 17, 19, 20, 21, 31, 36, 38, and 39.
- The article cited as Reference 5 was retracted during the peer review of [1]. The articles cited as References 2, 10, and 15 were retracted after [1] was published.

We regret that the issues were not identified prior to the article’s publication.

All authors either did not respond directly or could not be reached.
